# Do hypoechoic endometrial cystic appearances before embryo transfer affect pregnancy rates in frozen-thawed cycles?

**DOI:** 10.1371/journal.pone.0340292

**Published:** 2026-01-05

**Authors:** Ozge Karaosmanoglu, Nuri Peker, Burak Elmas, Aysen Yuceturk, Ilke Ozer Aslan, Omur Albayrak, Bulent Tiras

**Affiliations:** 1 Assisted Reproductive Technologies Unit, Acibadem Maslak Hospital, Istanbul, Türkiye; 2 Department of Obstetrics and Gynaecology, Acibadem Mehmet Ali Aydinlar University, Istanbul, Türkiye; ICAR-Indian Veterinary Research Institute: Indian Veterinary Research Institute, INDIA

## Abstract

**Background:**

The presence of hypoechoic cystic lesions (HCL) within the endometrium has been proposed as a potential factor influencing implantation and pregnancy outcomes during frozen–thawed embryo transfer (FET) cycles. However, evidence regarding their clinical significance remains limited and inconsistent. This study aimed to evaluate the effect of endometrial HCLs detected by transvaginal sonography on pregnancy and live-birth outcomes in women undergoing FET.

**Materials and Methods:**

This retrospective study included 88 women undergoing hormone replacement therapy–FET cycles, divided into two groups: patients with HCLs (*n* = 44) and controls with a normal trilaminar endometrial pattern (*n* = 44). The primary outcome was the live birth rate (LBR), which represents the most clinically meaningful endpoint of treatment success. Secondary outcomes included the clinical pregnancy rate (CPR) and miscarriage rate, while HCL size was analyzed as a secondary exploratory variable to assess potential associations with IVF outcomes.

**Results:**

There were no significant differences between groups in age, body mass index, duration of infertility, or endometrial thickness. The groups were also comparable regarding infertility cause, embryo-transfer day, and number of embryos transferred.

The LBR, the primary outcome, was 52.3% in the HCL group and 50.0% in controls (*p* = 0.670; OR = 1.10, 95% CI 0.47–2.53). Clinical pregnancy occurred in 68.2% of patients in both groups (*p* = 1.000), and biochemical pregnancy in 4.5% vs 2.3% (*p* = 1.000). Miscarriage rates were 20.5% and 15.9%, respectively (*p* = 1.000). No significant association was found between HCL size and any reproductive outcome.

**Conclusion:**

Hypoechoic cystic lesions within the endometrium do not appear to adversely affect pregnancy or live-birth outcomes in FET cycles when adequate endometrial thickness (≥7 mm) and a trilaminar pattern are present.

## Introduction

The endometrium, the inner lining of the uterus, is the site where an embryo implants and develops during pregnancy. It provides both structural anchorage and nourishment essential for early embryonic growth [1]. Successful live birth following in vitro fertilization (IVF) depends on two critical factors: the transfer of morphologically and genetically competent embryos, and a receptive endometrium capable of supporting implantation and continued development. Although several methods allow detailed evaluation of embryo quality, techniques for assessing endometrial receptivity remain limited [2].

Transvaginal sonography is the most widely used approach to evaluate endometrial thickness (ET) and endometrial pattern (EP) [2,3]. Numerous studies have demonstrated significant associations between ET and reproductive outcomes, including miscarriage and live birth rates (LBR). A threshold of approximately 7 mm has been frequently reported, below which pregnancy rates decline and miscarriage rates increase. In addition to thickness, the presence of a trilaminar EP is considered a critical indicator of receptivity, and is at least as important as the measured thickness itself [4–6]. Alterations in the echogenicity of the central line within this trilaminar pattern have been associated with reduced clinical pregnancy rates (CPR) [7].

Structural abnormalities such as intrauterine adhesions, polyps, and fibroids can distort the endometrial cavity, producing a dysmorphic uterine appearance that disrupts the EP and adversely affects pregnancy outcomes [8]. Furthermore, the occurrence of hypoechoic cystic lesions (HCL) during endometrial preparation has raised concern regarding impaired endometrial function and possible cycle cancellation. Even when embryo transfer proceeds, these lesions may indicate a reduced likelihood of pregnancy. The present study therefore aimed to evaluate the effect of HCL on pregnancy outcomes in patients undergoing frozen–thawed embryo transfer (FET).

## Materials and methods

This retrospective cohort study included FET cycles performed between January 2022 and January 2024 at the IVF Department of Acıbadem Maslak Hospital. Ethical approval was obtained from the Acıbadem Mehmet Ali Aydınlar University Medical Research Ethics Committee prior to data collection (*Approval No: 2025-06/257*). As anonymized data from existing medical records were used, the requirement for individual informed consent was waived in accordance with institutional and national regulations.

### Study population

Patients were classified into two groups according to endometrial appearance on transvaginal ultrasonography: those with HCL (HCL group) and those with a normal trilaminar EP (control group). Only the first FET cycle per patient was analyzed to eliminate potential bias from repeated treatments. All included cases had an ET of ≥7 mm. Exclusion criteria included maternal age > 40 years, a history of recurrent implantation failure or recurrent pregnancy loss, uterine anomalies such as intrauterine adhesions or dysmorphic uterus, and male partners with azoospermia. Patients with previous uterine surgery (myomectomy, septum resection, or adhesiolysis) or sonographic evidence of intrauterine adhesions, endometrial polyps, or fibroids were excluded to prevent confounding effects on endometrial morphology and implantation outcomes.

### Endometrial preparation and embryo transfer protocol

All patients received a gonadotropin-releasing hormone (GnRH) agonist (Lucrin Depot 3.75 mg/0.1 mL, leuprolide acetate) on day 21 of the menstrual cycle preceding endometrial preparation. On cycle days 2–3, transvaginal sonography was performed to evaluate the endometrial cavity and adnexa, and serum estradiol and progesterone levels were measured. Oral estrogen (Estrofem 2 mg) was given twice daily for the first five days, three times daily for the next five days, and four times daily thereafter. On day 15 of estrogen administration, patients were reevaluated. No HCL was observed at baseline (cycle days 2–3); these lesions were first identified during sonographic evaluation on day 15, coinciding with the measurement of ET.

Cycles were cancelled if ET was < 7 mm. For patients with adequate endometrial development (≥7 mm), serum progesterone and estradiol levels were reassessed. Those with progesterone levels <1.0 ng/mL and estradiol levels >200 pg/mL initiated progesterone supplementation to optimize endometrial–embryo synchronization and prevent premature luteinization, consistent with the findings of Yuceturk *et al*. (2025), who demonstrated that serum hormone levels at specific time points are predictive of FET outcomes [9]. Luteinizing hormone levels were not measured, as all patients were under complete GnRH-agonist suppression. Blood samples were collected between 08:00 and 10:00 on both the day of endometrial assessment and the day of embryo transfer.

Luteal support consisted of subcutaneous prolutex (25 mg twice daily) combined with vaginal progesterone capsules (Progestan 200 mg three times daily). Embryo transfer timing was based on the duration of progesterone exposure: cleavage-stage embryos were transferred after three full days (P + 3) and blastocysts after five full days (P + 5). To ensure uniform luteal support, patients with serum progesterone levels <14.97 ng/mL on the day of transfer were excluded. Only FET cycles without preimplantation genetic testing (PGT) were included to eliminate bias related to embryo chromosomal status, and all transfers were performed as single-embryo transfer (SET) to maintain consistency between groups.

### Outcomes

Pregnancy outcomes were defined according to standard clinical criteria. A biochemical pregnancy was defined as a positive serum β-hCG (>10 mIU/mL) without sonographic evidence of a gestational sac. A clinical pregnancy was defined as the visualization of an intrauterine gestational sac with fetal cardiac activity at approximately 5–6 weeks of gestation. Miscarriage referred to any pregnancy loss occurring before 25 weeks of gestation, including biochemical losses. Live birth was defined as the delivery of a liveborn infant at or beyond 25 weeks of gestation. No ectopic pregnancies were identified, and biochemical and clinical pregnancies were both included in the denominators for clinical pregnancy and live-birth rate calculations. The primary outcome was the LBR, while secondary outcomes included the CPR, miscarriage rate, and biochemical pregnancy rate. The size of HCLs was further analyzed as an exploratory variable to assess potential correlations with reproductive outcomes.

### Statistical analysis

Statistical analyses were performed using IBM SPSS Statistics version 30.0 (IBM Corp., Armonk, NY, USA). Data normality was assessed with the Shapiro–Wilk test. Normally distributed continuous variables were expressed as mean ± standard deviation (SD), and non-normally distributed variables as median [interquartile range, IQR]. Categorical variables were presented as number (*n*) and percentage (%). Between-group comparisons for continuous variables were conducted using the independent-samples t-test for normally distributed data and the Mann–Whitney U test for non-normally distributed data. Categorical variables were compared using the Pearson chi-square or Fisher’s exact test when more than 20% of expected cell counts were <5. Effect sizes for binary outcomes were reported as odds ratios (OR) with 95% confidence intervals (CI). For multi-category variables, such as embryo-transfer pattern and cause of infertility, only overall χ² p-values were reported to minimize multiplicity and unstable estimates. Associations between continuous variables, such as hypoechoic lesion size and reproductive outcomes, were evaluated using the Mann–Whitney U test. A two-tailed *p* value <0.05 was considered statistically significant.

### Results

**Fig 1:**
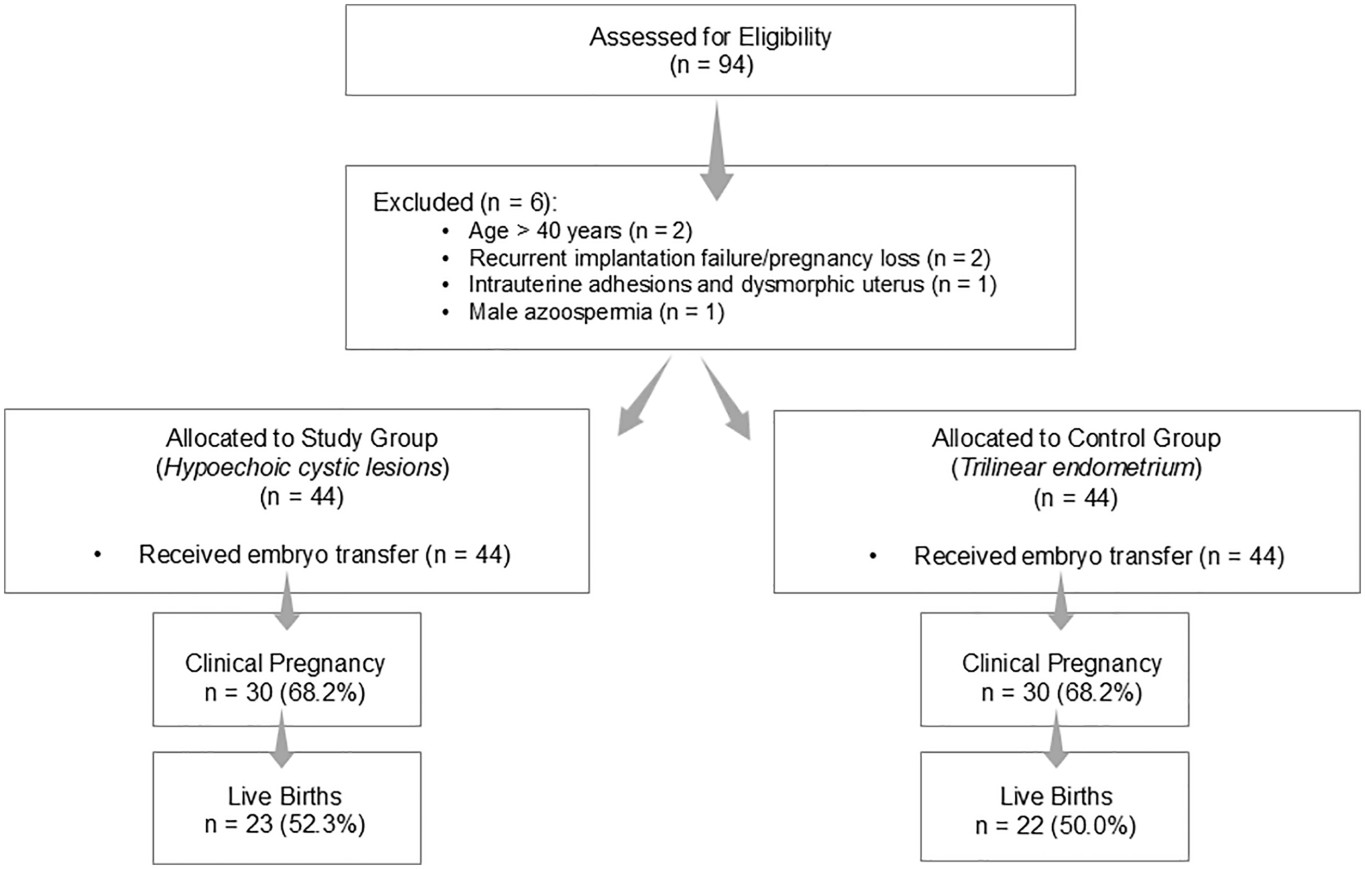
Study flow diagram of patient selection and outcomes.

A total of 88 patients were included in the analysis, comprising 44 patients with HCLs (HCL group) and 44 controls with a normal trilaminar EP (**Fig 1**). The mean age of all participants was 32.5 ± 6.2 years, with a mean body mass index (BMI) of 26.8 ± 6.7 kg/m^2^, mean ET of 11.1 ± 2.3 mm, and mean infertility duration of 5.7 ± 4.0 years (**Table 1**). When compared by group, no significant differences were observed in age (32.8 ± 6.3 vs 32.2 ± 6.1 years, *p* = 0.74), BMI (27.1 ± 6.5 vs 26.6 ± 6.9 kg/m^2^, *p* = 0.81), ET (11.2 ± 2.4 vs 11.0 ± 2.3 mm, *p* = 0.72), or duration of infertility (5.8 ± 3.9 vs 5.6 ± 4.2 years, *p* = 0.84). Effect sizes were uniformly small, indicating well-matched baseline characteristics. In the HCL group, the median lesion diameter was 0.9 mm [IQR 0.3–1.8], suggesting that the observed lesions were typically small and localized.

**Table 1:** Demographic and baseline characteristics of the study population.

Variable	Overall (n = 88)	HCL Group (n = 44)	Control Group (n = 44)	*p* value	Cohen’s *d*
Age (years)	32.5 ± 6.2	32.8 ± 6.3	32.2 ± 6.1	0.74	0.10
Body Mass Index (kg/m²)	26.8 ± 6.7	27.1 ± 6.5	26.6 ± 6.9	0.81	0.07
Endometrial thickness (mm)	11.1 ± 2.3	11.2 ± 2.4	11.0 ± 2.3	0.72	0.09
Duration of infertility (years)	5.7 ± 4.0	5.8 ± 3.9	5.6 ± 4.2	0.84	0.05
Diameter of hypoechoic cystic lesions (mm) †	0.9 [0.3–1.8]	0.9 [0.3–1.8]	—	—	—

Values are presented as mean ± standard deviation (SD) or median [IQR] where appropriate. Cohen’s d represents standardized effect size (small = 0.2, medium = 0.5, large = 0.8). † Data reported for patients with detectable hypoechoic cystic lesions only (HCL group).

The LBR was the primary outcome, while the CPR, biochemical pregnancy rate, and miscarriage rate were analyzed as secondary outcomes (**Table 2**). The size of HCLs was examined as an exploratory variable. Clinical pregnancy was achieved in 30 patients (68.2%) in both groups (*p* = 1.00; OR = 1.00, 95% CI 0.41–2.46). Biochemical pregnancy occurred in 2 (4.5%) and 1 (2.3%) cases in the HCL and control groups, respectively (*p* = 1.00; OR = 2.05, 95% CI 0.18–23.44). Miscarriage occurred in 5 (20.5%) and 7 (15.9%) patients (*p* = 1.00; OR = 0.68, 95% CI 0.20–2.33), while LBRs were 23 (52.3%) and 22 (50.0%) in the HCL and control groups, respectively (*p* = 0.67; OR = 1.10, 95% CI 0.47–2.53). None of these differences were statistically significant, indicating that the presence of HCLs did not adversely influence pregnancy or live-birth outcomes following FET. Further analysis showed no significant association between HCL size and reproductive outcomes. The median diameter was 1.80 mm [IQR 1.20–2.00] in patients who conceived and 1.65 mm [IQR 1.30–3.00] in those who did not (*p* = 0.612). Similarly, lesion size did not differ between patients with and without live birth (*p* = 0.795) (**Table 2**).

**Table 2:** Comparison of reproductive outcomes between patients with and without hypoechoic cystic lesions (HCLs).

Outcome	HCL Group (n = 44)	Control Group (n = 44)	*p* value	OR (95% CI)
Live birth, n (%)	23 (52.3%)	22 (50.0%)	0.67	1.10 (0.47–2.53)
Clinical pregnancy, n (%)	30 (68.2%)	30 (68.2%)	1	1.00 (0.41–2.46)
Biochemical pregnancy, n (%)	2 (4.5%)	1 (2.3%)	1	2.05 (0.18–23.44)
Miscarriage, n (%)	5 (20.5%)	7 (15.9%)	1	0.68 (0.20–2.33)
***HCL size and reproductive outcomes***				
**Outcome comparison**	**Group 1**	**Group 2**	**Median HCL size (mm) [IQR]**	***p* value**
Pregnancy status	Pregnant (n = 30)	Non-pregnant (n = 14)	1.80 [1.20–2.00]	0.612
Live-birth status	Live birth (n = 23)	No live birth (n = 21)	1.65 [1.30–3.00]	0.795

*Number of patients with evaluable data.

The distribution of embryo-transfer patterns was identical between groups (χ² *p* = 1.000) (**Table 3**). In both cohorts, 5 (11.4%) patients received one embryo on day 3, 14 (31.8%) received one embryo on day 5, 6 (13.6%) received two embryos on day 3, and 19 (43.2%) received two embryos on day 5. Per-category odds ratios were 1.00 with wide confidence intervals (e.g., one embryo on day 5: OR = 1.00, 95% CI 0.44–2.28), confirming comparable embryo-transfer distributions between groups. Infertility etiologies were comparable between groups (χ² *p* = 0.444) (**Table 3**). In the HCL group, male-factor, diminished ovarian reserve, advanced maternal age, and unexplained infertility were present in 43.2%, 6.8%, 13.6%, and 36.4% of patients, respectively, with no tubal-factor cases. Corresponding rates in the control group were 38.6%, 11.4%, 2.3%, and 38.6%, with 9.1% having tubal-factor infertility. None of the differences were statistically significant (all *p* > 0.05), indicating that HCLs were not associated with the underlying infertility profile.

**Table 3:** Embryo-transfer characteristics and causes of infertility in patients with and without hypoechoic cystic lesions (HCL).

Parameter	HCL Group	Control Group	*p* value
	(n = 44)	(n = 44)	
**Embryo-transfer pattern (*overall χ² p = 0.214*)**			
1 embryo, Day 5	16 (36.4%)	17 (38.6%)	0.875
1 embryo, Day 3	1 (2.3%)	0 (0%)	1
2 embryos, Day 5	24 (54.5%)	25 (56.8%)	0.841
2 embryos, Day 3	2 (4.5%)	2 (4.5%)	1
3 embryos, Day 5	1 (2.3%)	0 (0%)	1
Embryo quality (mean grade ± SD)	3.5 ± 0.6	3.6 ± 0.5	0.587
Number of embryos transferred (mean ± SD)	1.1 ± 0.3	1.1 ± 0.3	0.914
**Cause of infertility**			
Male factor	19 (43.2%)	17 (38.6%)	0.673
Tubal factor	0 (0%)	4 (9.1%)	0.116
Diminished ovarian reserve	3 (6.8%)	5 (11.4%)	0.712
Advanced maternal age	6 (13.6%)	1 (2.3%)	0.114
Unidentified infertility	16 (36.4%)	17 (38.6%)	0.841

Overall, the presence or size of HCLs did not influence implantation, clinical pregnancy, or live-birth outcomes following FET, suggesting that small, localized endometrial lesions are clinically insignificant in terms of reproductive prognosis.

## Discussion

Endometrial preparation is a critical determinant of success in IVF, as it directly influences implantation and conception outcomes. Even minor variations in endometrial characteristics can significantly affect reproductive success. Among the parameters used to assess endometrial suitability, thickness and pattern are the most widely studied. Transvaginal sonography remains the primary modality for evaluating the endometrium before embryo transfer and facilitates the detection of intrauterine pathologies such as polyps, fibroids, fluid accumulation, and hypoechoic lesions. Additionally, uterine peristalsis has been shown to influence IVF outcomes [10], while Doppler sonography provides valuable insights into endometrial receptivity by assessing subendometrial blood flow [3].

Endometrial thickness (ET) has long been established as a major predictor of implantation and live birth following IVF. Studies have reported pregnancy rates across a wide range of ET values, from <4 mm to >15 mm, with CPRs varying between 9.1% and 79.3% depending on patient and cycle characteristics [11,12]. Notably, an ET of less than 7 mm is consistently associated with lower pregnancy rates and higher rates of biochemical loss or miscarriage [11]. Von Wolff *et al*. reported pregnancy rates of 7.4% in patients with ET < 7 mm compared with 30.8% in those with ET > 7 mm [13]. Likewise, Holden *et al*. observed significantly higher LBRs in patients with ET ≥ 11 mm than in those with 7–11 mm [14], and Richter *et al*. found that ET ≥ 16 mm was associated with markedly higher CPR than ET < 9 mm (77% vs. 53%) [15]. Collectively, these studies highlight the importance of ET as a key determinant of implantation potential and live birth.

Beyond thickness, the EP also serves as a critical indicator of receptivity. A trilaminar pattern is traditionally regarded as the most receptive configuration [16]. Zhao *et al*. demonstrated that patients with ET between 7 and 14 mm and a trilaminar EP achieved the most favorable IVF outcomes [16]. Likewise, Kuramoto *et al*. classified EP into three categories—leaf, partial-leaf, and non-leaf—and found that non-leaf patterns were associated with significantly lower pregnancy rates [7]. Conversely, Cheng *et al*. proposed an alternative classification system and reported that deviations from the trilaminar pattern were linked to increased rates of early pregnancy loss and lower LBR [2]. Although a few studies have presented contradictory findings, most evidence supports the association between a trilaminar EP and higher CPR.

Another factor influencing IVF outcomes is intrauterine fluid accumulation, which has been associated with reduced implantation rates. While Zhang *et al*. reported lower CPR in patients with intrauterine fluid [17], other studies suggested that fluid aspiration before transfer mitigates adverse effects [18–20]. These findings emphasize that both endometrial structure and cavity integrity are key prerequisites for successful implantation.

Hypoechoic cystic lesions represent a distinct form of endometrial abnormality with potential clinical relevance. These lesions, characterized by one or more well-defined hypoechoic cystic areas within the endometrium on ultrasound, vary in size and margin clarity but typically lack internal or peripheral vascularization [21–23]. Because of their sonographic similarity, they may be misdiagnosed as myomas, which are hypoechoic but exhibit acoustic shadowing, or as endometrial hyperplasia, which often appears as cystic areas with endometrial thickening [22]. Fluid accumulation may present a similar appearance, necessitating careful differential diagnosis before embryo transfer to exclude conditions that could impair endometrial receptivity and implantation.

Published data on the clinical impact of HCLs remain scarce. To date, this is only the second study to specifically assess their effect on IVF outcomes. Tian *et al*. reported lower LBR and a higher incidence of first-trimester miscarriage in patients with HCLs compared with those without, but found no association between lesion size and clinical outcomes [21]. In contrast, our findings indicate that the presence of small, localized cystic lesions does not adversely affect pregnancy or LBR. In our cohort, all patients had adequate ET (≥7 mm), a trilaminar pattern, and no intrauterine fluid accumulation—conditions reflecting optimal endometrial receptivity. These factors may explain why HCL did not impair implantation or reproductive success in our study.

In conclusion, when the endometrium exhibits sufficient thickness and a normal trilaminar pattern, the presence of small cystic lesions appears to have no detrimental impact on pregnancy or live birth outcomes. Our findings align with the hypothesis that, under optimal endometrial preparation, such lesions are incidental findings rather than indicators of impaired receptivity.

## Limitations

The main limitation of this study is its retrospective design, which may involve selection bias and incomplete data capture. The relatively small sample size (44 patients per group) provided adequate power only to detect large differences in live-birth rates; smaller effects cannot be excluded. Although all scans were performed by experienced clinicians, some misclassification between HCL and other intra-endometrial findings (e.g., fluid, polyps, focal hyperplasia) is possible. The lack of centralized or blinded image review may have introduced observer variability, and unmeasured factors such as mild adenomyosis could have influenced outcomes. Serum progesterone on the transfer day was not measured, limiting assessment of luteal adequacy. Larger prospective studies with standardized imaging and hormonal monitoring are needed to confirm these results.

## Conclusion

This study evaluated the impact of cystic hypoechoic lesions on IVF outcomes and found that their presence did not adversely affect clinical pregnancy or LBR in patients with adequate ET (>7 mm) and a trilaminar pattern. However, given the limited sample size, larger prospective studies are warranted to confirm these findings and further elucidate the clinical relevance of such lesions in endometrial assessment before embryo transfer.

## Supporting information

S1 Table Individual-level clinical, treatment, and outcome data of patients included in the study. (CSV)
